# Immunization of cervidized transgenic mice with multimeric deer prion protein induces self-antibodies that antagonize chronic wasting disease infectivity *in vitro*

**DOI:** 10.1038/s41598-017-11235-8

**Published:** 2017-09-05

**Authors:** Dalia H. Abdelaziz, Simrika Thapa, Basant Abdulrahman, Li Lu, Shikha Jain, Hermann M. Schatzl

**Affiliations:** 1 Department of Comparative Biology & Experimental Medicine, University of Calgary, Calgary, Alberta Canada; 2 Department of Biochemistry and Molecular Biology, Faculty of Pharmacy, Helwan University, Cairo, Egypt; 3 Calgary Prion Research Unit, University of Calgary, Calgary, Alberta Canada

## Abstract

Chronic wasting disease (CWD) is the most contagious prion disease. It is expanding rapidly in North America, was found recently in Europe, and the potential for transmission to humans cannot be excluded yet. We hypothesized that it is possible to prevent peripheral CWD infection and CWD prion shedding by inducing auto-antibodies against the cellular prion protein (PrP^C^) by active vaccination. Our objective is to overcome self-tolerance against PrP by using a multimeric recombinant PrP (recPrP) as an immunogen. We expressed in *E. coli*, purified and refolded four immunogens: cervid and murine recPrP in monomeric and dimeric form. Testing immunogenicity in sera of the vaccinated transgenic mice expressing cervid PrP revealed that all four immunogens effectively overcame self-tolerance against the prion protein as shown by high antibody titers. Confocal microscopy analysis revealed effective binding of post-immune sera to surface-located PrP^C^ in both murine and cervid PrP expressing cultured cells. Remarkably, the post-immune auto-antibodies effectively inhibited CWD-induced prion conversion in RT-QuIC assay when incubated with either PrP substrate or CWD seed. Furthermore, they mitigated prion propagation in CWD-infected cervid-PrP expressing RK13 cells. Together, multimeric recombinant cervid PrP effectively overcomes self-tolerance to PrP and induces auto-antibodies that interfere with CWD conversion *in vitro*.

## Introduction

Chronic wasting disease (CWD) is a rapidly expanding fatal transmissible spongiform encephalopathy (TSE) or prion disease affecting free-ranging and captive cervids (deer, elk and moose). CWD is present in North America, South Korea and recently was found in Northern Europe as well^1, 2^. CWD is considered the most contagious prion disease. The rapid spread of CWD is attributed to effective animal-to-animal transmission as well as pronounced environmental contamination by shedding of prions in excreta like feces, saliva, urine, and infected carcasses^3–6^. The effective horizontal transmission, the long-term presence of infectivity in the environment, and the occurrence in wild-ranging animals, makes containment and control of CWD truly challenging. Neither prophylactic vaccines nor effective treatment or containment options are presently available^7^. In addition, the zoonotic potential of CWD cannot be excluded yet^1^.

There is a solid body of evidence that passive and active immunization has the potential to block prion propagation in murine-adapted scrapie *in vivo* models^8–12^. The major challenge in active vaccination strategies against prion diseases is the tolerance against the prion protein^10, 11^. Prions use conformational conversion of the endogenous cellular prion protein (PrP^C^) into the pathological isoform PrP^Sc^ for their propagation^13^. Whenever prions are generated *de novo* in an infected human or animal, they represent the species of the host, and adaptive immunity based on MHC-presentation of linear epitopes is not able to discriminate the two isoforms^14^.

Very few studies have reported active vaccination against CWD. One study used a synthetic peptide vaccine approach and did not show any success in protection of mule deer against CWD^15^. A more recent study demonstrated partial protection of white-tailed deer against CWD^16^. In this study, deer were immunized orally with an attenuated *Salmonella typhimurium* vector vaccine expressing cervid PrP. Despite the vaccination protocol used was very extensive and included also prime-boost regimens with recombinant PrP vaccine formulations, the obtained antibody titers were very low and the protection rate was only around 20%^16^. Nonetheless, this study provides a solid proof-of-concept that active vaccination has the potential to interfere in CWD infection.

In the current study, our objective was to overcome self-tolerance against cervid PrP by using a multimeric recombinant cervid PrP as an immunogen. Induced self-antibodies should bind to PrP^C^ on the surface of cells, thereby blocking the substrate for cellular prion replication and interfere with the peripheral propagation of prions. As our previous experimental data in wild-type mice have shown, this approach induces a robust humoral immunity against PrP^C^ without detectable side effects^10, 17^, and is able to protect some immunized mice^11^. Having a solid proof-of-concept for our self-vaccination approach in wild-type mice, led us to test whether multimeric cervid PrP can be used for active vaccination against CWD in a transgenic mouse model susceptible to CWD prions.

## Results

### Vaccination of tg(cerPrP)1536^+/+^ mice with deer recombinant PrP induces anti-PrP auto-antibodies

**Figure 1:**
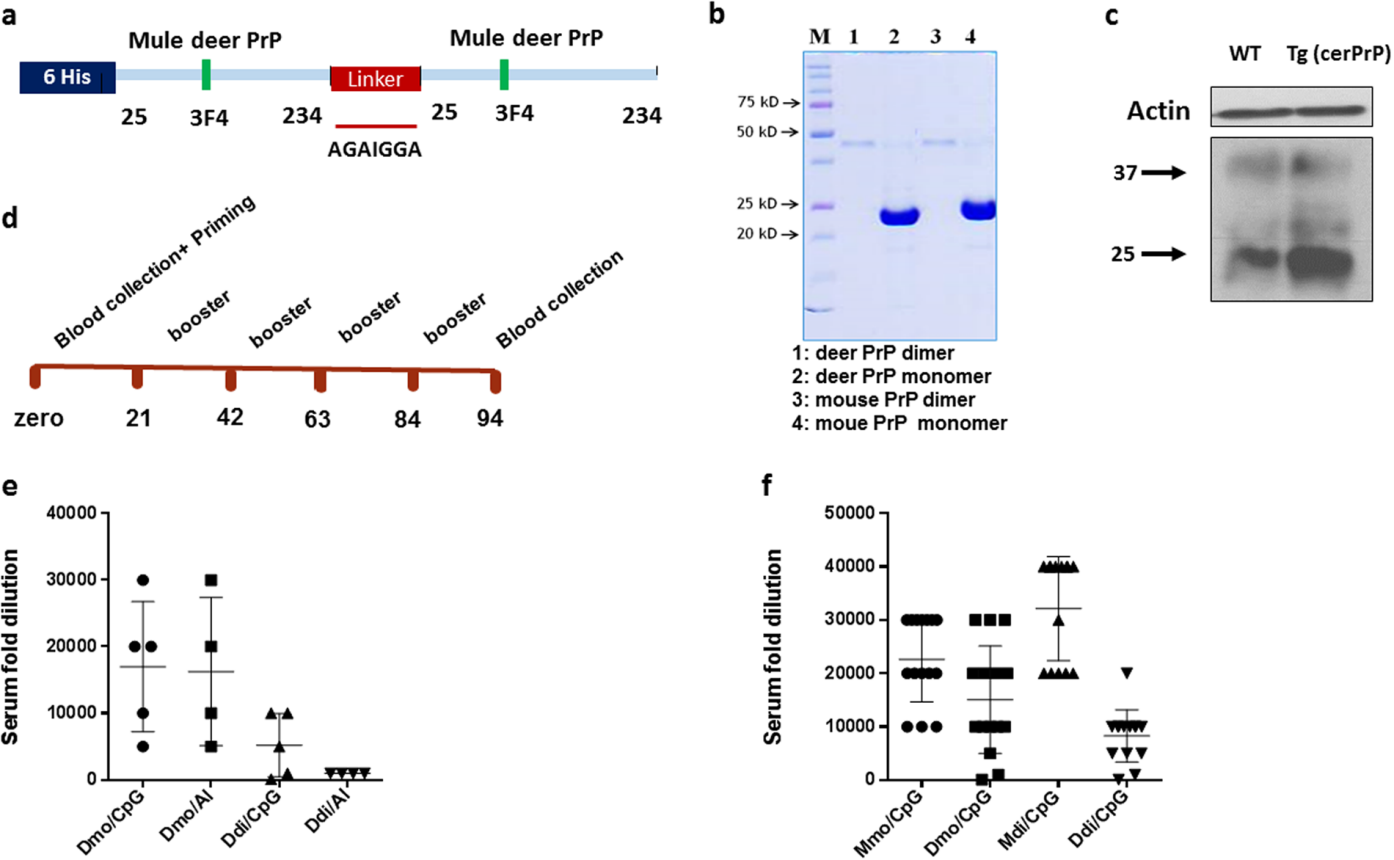
Immunization of tg (cerPrP) 1536^+/+^ mice with recombinant prion proteins (rPrP) induces high auto-antibodies titers. (**a**) Deer dimeric recombinant prion protein was constructed by covalently linking 2 mature PrP moieties with a 7 amino acid linker. The construct contains epitope tags for mAb 3F4 and has an N-terminal polyhistidine tag for purification. (**b**) Proteins were expressed in *E*. *coli*, purified under denaturing conditions using IMAC technology, and refolded by dialysis. Purity of immunogens was tested by SDS-PAGE followed by Coomassie blue staining. (**c**) PrP^C^ expression in brain homogenates of uninfected wild-type C57 BL/6 (WT) and transgenic mice overexpressing deer PrP [Tg (cer PrP)] was analysed in immunoblot (mAb 4H11), using actin as a loading control. (**d**) Tg (cerPrP) 1536^+/+^ mice were immunized 5 times with immunogens in 3 weeks’ intervals and blood sampling was performed either before starting vaccination or 10 days after the last booster dose. (**e**) Antibody titers were determined using end-point ELISA. Immunogens were either deer monomeric (Dmo) or deer dimeric recombinant PrP (Ddi), either with alum (Dmo/Al, Ddi/Al) or CpG as adjuvant (Dmo/CpG, Ddi/CpG). Antibody titers for individual mice were determined by end-point dilution. The y-axis indicates the serum fold dilution. Cut-off was calculated as 3 times average OD of pre-immune sera. (**f**) Antibody titers using end point ELISA the four vaccinated groups (15 mice each). Mice were vaccinated with Mmo, Mdi, Dmo, or Ddi recombinant PrPs, and CpG was used as adjuvant for all groups. The antibody titers for the individual mouse were determined by end-point dilution. The y-axis indicates the serum fold dilution. The cut-off was calculated as 3 times average OD of pre-immune sera.

To overcome the self-tolerance to cervid PrP in tg(cerPrP)1536^+/+^ mice, we used a recombinant PrP (rPrP) dimer as immunogen, in which two moieties of deer PrP (residues 25-234) were linked covalently by seven amino acid (aa) linker (Ddi; Fig. 1a). In addition, a monomeric version of recombinant deer PrP (Dmo) was used. These immunogens represent self-antigens in the immunized deer-PrP expressing transgenic mice. As a non-self control, we used murine dimeric and monomeric rPrP (Mdi, Mmo), as described previously^10^. Mouse and deer PrP differ by 21 amino acids (~10%; mouse PrP 23–231) (Fig. S1)^18, 19^. The four immunogens (Mmo, Mdi, Dmo and Ddi) were expressed as recombinant proteins in *E. coli*, purified under denaturing conditions using immobilized metal ion affinity chromatography (IMAC), and refolded by dialysis overnight. Coomassie staining of SDS-PAGE showed that the yield of monomeric immunogens (Dmo and Mmo) was greater than that of dimeric ones (Ddi and Mdi), however all immunogens were of high purity (Fig. 1b). The transgenic mice (tg(cerPrP)1536^+/+^) are over-expressing deer PrP and have been used widely for investigating CWD^20–22^. The expression level of cervid PrP in brain homogenate of tg(cerPrP)1536^+/+^ mice is higher than that of mouse PrP in brain homogenate of wild-type C57BL/6 mice (Fig. 1c), representing a potential obstacle in overcoming the self-tolerance to PrP. The vaccination schedule used throughout the study is shown in Fig. 1d.

To test the efficacy of different adjuvants we conducted a small-scale study in which we used alum and type B CpG oligonucleotide (CpG) as adjuvants with self-antigens (Dmo and Ddi). Ten days after the fourth booster dose serum was collected from each mouse and analyzed for antibody titers using end-point dilution ELISA. We found that CpG was a better adjuvant for Ddi rPrP than alum, with no differences for Dmo rPrP (Fig. 1e). On a larger scale immunization experiment we then tested the four immunogens (Mmo, Mdi, Dmo and Ddi) using CpG as adjuvant. Mdi induced the highest titers, followed by Mmo, as expected for non-self immunogens. Dmo was better than Ddi rPrP, yielding the lowest titers (Fig. 1f). There was greater inter-individual variability within deer rPrP-immunized groups and very few mice did not react. Of note, the average titer for the Ddi rPrP vaccinated group was still reasonably high (1:10,000) (Fig. 1f).

In summary, these data indicate that all immunogens efficiently break the tolerance to PrP and produce high titers of anti-PrP antibodies in ELISA.

### Post-immune sera bind effectively to authentic PrP^C^ and PrP^Sc^

**Figure 2:**
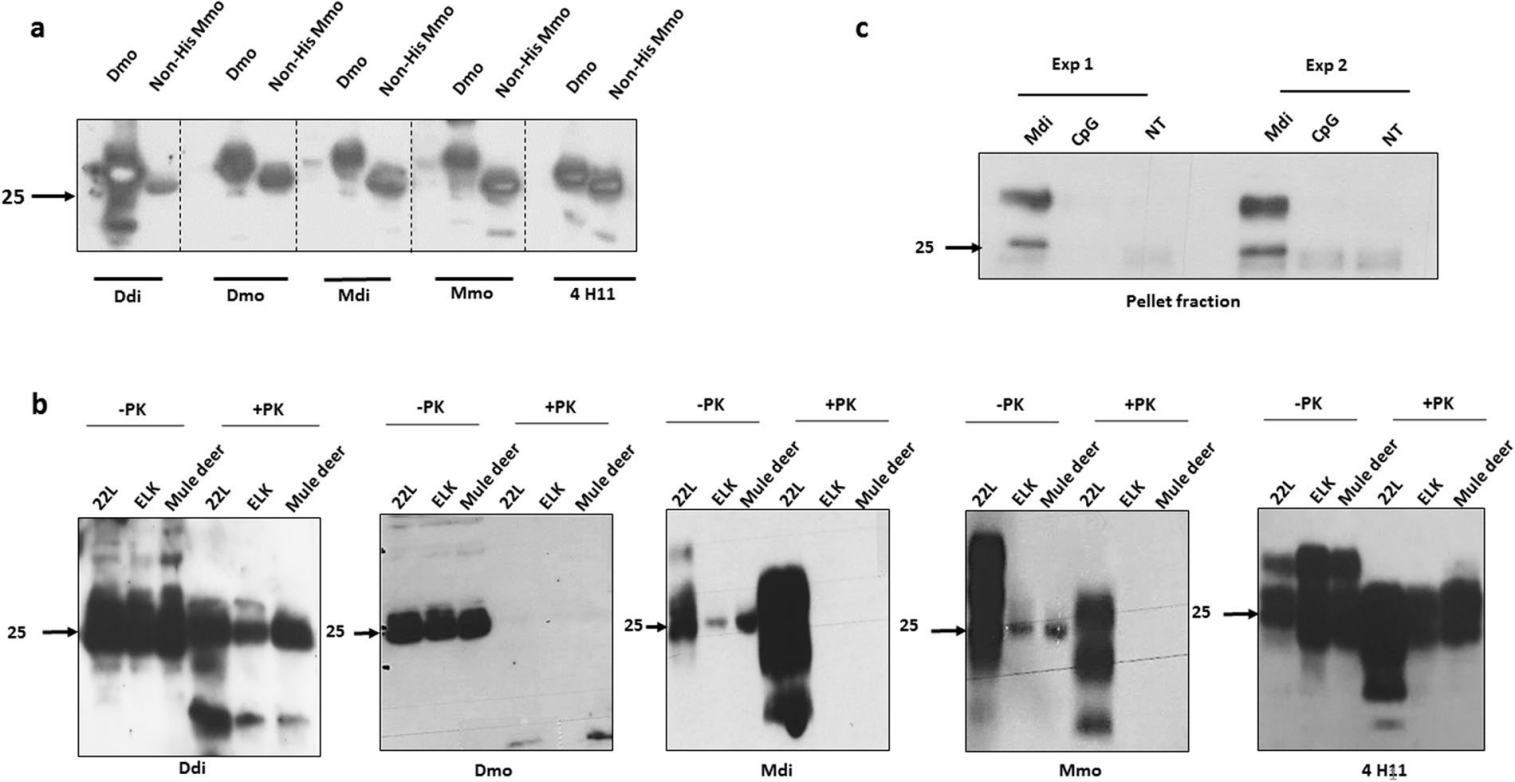
Post-immune sera detect recombinant, native and proteinase K resistant PrP. (**a**) Recombinant deer monomeric (Dmo) and non-His-tagged mouse monomeric recombinant PrP (non-His Mmo) were loaded on SDS PAGE and immunoblotted with post-immune sera (1:250 dilution) of mice immunized with Ddi, Dmo, Mdi and Mmo recombinant PrPs. mAb 4H11 was used as a control. (**b**) Brain homogenates (10%) from wild-type mice infected with 22 L, or tg mice infected with elk or mule deer CWD were subjected, or not, to proteinase K (PK) digestion (50 µg/ml for 1 hr) and then samples subjected to immunoblot analysis. As primary antibodies, post-immune sera of mice vaccinated with Ddi, Dmo, Mdi or Mmo recombinant PrPs, all in 1:500 dilution, were used. The mAb 4H11 (1:500) was employed as control antibody. (**c**) N2a cells were treated for 4 days, or not (NT), with post-immune sera from mice immunized with Mdi recombinant PrP (1:100), sera from adjuvant-only (CpG) treated mice were used as control (1:100). Then cells were lysed, cell lysates treated with 1% sarcosyl and ultracentrifuged at 100,000 g for 1 hour. The pellet fractions were immunoblotted with anti-PrP polyclonal rabbit antibody (pAb) 142.

To investigate the efficiency of post-immune antibodies in binding to recombinant full-length PrP in immunoblot we used monomeric deer (Dmo) recombinant PrP. Furthermore, we used non-HIS-tagged mouse monomeric rPrP (non-his Mmo), to exclude that post-immune sera are reactive only against the poly-histidine tag. We found that post-immune sera from all vaccinated groups efficiently detected rPrP when used as primary antibody in immunoblots (Fig. 2a). Of note, post-vaccination sera of Ddi-immunized mice showed the strongest signal for detection of Dmo (self), and the weakest for Mmo rPrP (non-self). Given that we used the same dilution (1:250) for all post-immune sera, Ddi-immunized sera revealed the highest reactivity to deer PrP despite being the most diluted one because of its low ELISA titer. To test the ability of post-immune sera to detect natural PrP and PrP^Sc^, we used 10% brain homogenate (BH) of terminally ill mice infected with 22 L, elk CWD or mule deer CWD as antigen on immunoblots. Brain homogenate was subjected or not to PK digestion (−PK/+PK). The immunoblots in which the post-vaccination sera were used as primary antibodies revealed that only post-immune sera from Ddi-vaccinated mice could detect total PrP (−PK) and PrP^Sc^ (+PK) of mule deer CWD, elk CWD and mouse 22 L (Fig. 2b). Post-immune sera from Dmo-vaccinated mice could detect only total PrP in all three brain homogenates (Fig. 2b). Sera from Mdi and Mmo- vaccinated groups effectively detected total PrP and PrP^Sc^ of 22 L brain homogenates, with minor signals for deer and elk total PrP. Remarkably, the signal detected by post-immune sera of Mdi and Mmo-vaccinated mice were comparable to the signals detected with the monoclonal antibody 4H11 used as a positive control. CpG-only sera were negative (data not shown).

There is consensus in the prion field that antibodies have to effectively bind to naturally folded and authentic PrP^C^ on the surface of cells to be protective^11, 23^. To characterize the affinity of post-immune sera to native PrP^C^, we applied sera of mice vaccinated with Mdi to the media of cultured N2a cells in order to test whether the antibodies are binding to surface-located authentic PrP^C^ and induce PrP aggregation by cross-linking as seen before^10, 17^. Cell lysates were subjected to a detergent solubility assay and pellet fractions analyzed in immunoblot (Fig. 2c). We found a pronounced insoluble PrP^C^ fraction in the pellet of Mdi-sera treated cells, whereas the insoluble fractions were negligible in cells not treated with sera or treated with control (CpG-only) post-immune sera.

These data confirm that the antibodies in the post-immune sera are able to sequester and crosslink native PrP^C^, diminishing its detergent solubility.

### Post-immune sera bind to mouse and deer surface PrP^C^ in cultured cells

**Figure 3:**
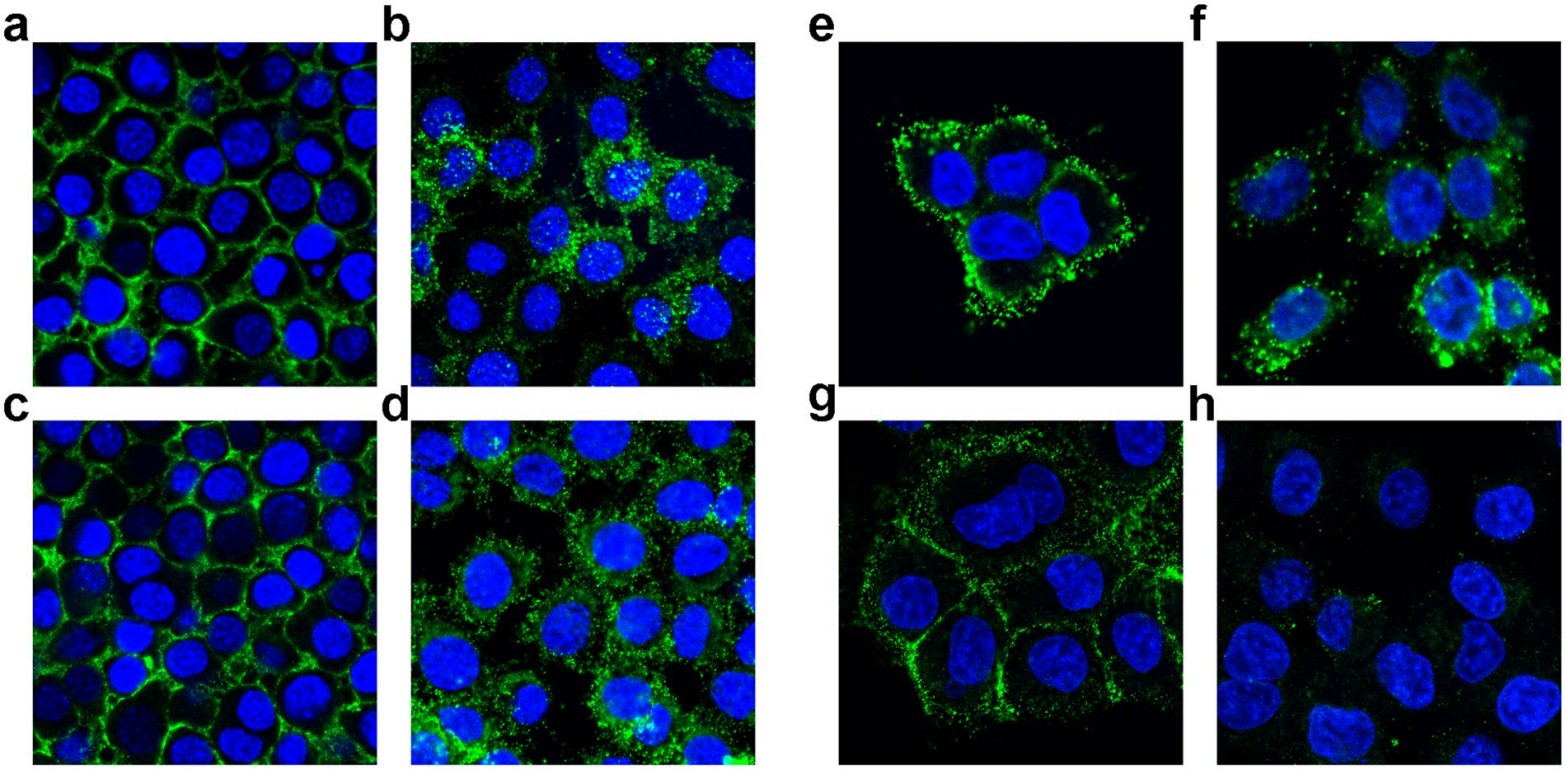
Post-immune sera bind to surface-located PrP^C^ on N2a cells and RK13 cells, overexpressing cervid PrP^C^, and are internalized. Confocal microscopy analysis for binding and internalization of post-immune sera from Mdi vaccinated mice (**a**,**b**) and mAb 4H11 (**c**,**d**) in N2a cells. Cultured N2a cells were incubated at 4 °C (**a**,**c**) and analyzed for testing membrane binding, or analyzed after 60 min incubation at 37 °C (**b**,**d**) for internalization. Confocal microscopy analysis for binding and internalization of post-immune sera from Ddi (**e**), mAb 4H11(**g**), and the control CpG (**h**) labeling cell surface PrP^C^ from RK13 cells overexpressing cervid PrP^C^ at 4 °C, Ddi serum were internalized into cer-RK13 at 37 °C following surface binding (**f**).

For further characterization of the affinity of post-immune sera to native PrP^C^, uninfected N2a cells treated with Mdi post-immune sera were examined by confocal microscopy. Post-immune sera bound to surface PrP^C^ after treatment of the cells at 4 °C, with intensity similar to that of the monoclonal antibody 4H11 used as control (Fig. 3a,c). The PrP^C^–antibody complex was effectively internalized when assayed after 60 min of incubation at 37 °C, resulting in a perinuclear staining with similar intensity for post-immune sera and mAb 4H11 (Fig. 3b,d). This provides additional evidence for the specific binding of post-immune sera to natural PrP^C^.

As our main target is deer PrP^C^, we tested the binding and internalization of antibodies in post-immune sera from Ddi-immunized mice in cervid PrP expressing RK13 cells (Fig. 3e,f). mAb 4H11 was used as control antibody (Fig. 3g), with anti-mouse secondary antibodies. As before, there was a pronounced surface staining and internalization to perinuclear compartments, indicating specific binding of antibodies to surface-located cervid PrP^C^. Sera from CpG-only vaccinated mice did not show any surface binding (Fig. 3h).

Taken together, these data demonstrate that self-antibodies induced in post-immune sera effectively bind and crosslink surface cervid PrP^C^ in RK13 cells.

### Self-antibodies block CWD prion conversion in RT-QuIC

**Figure 4:**
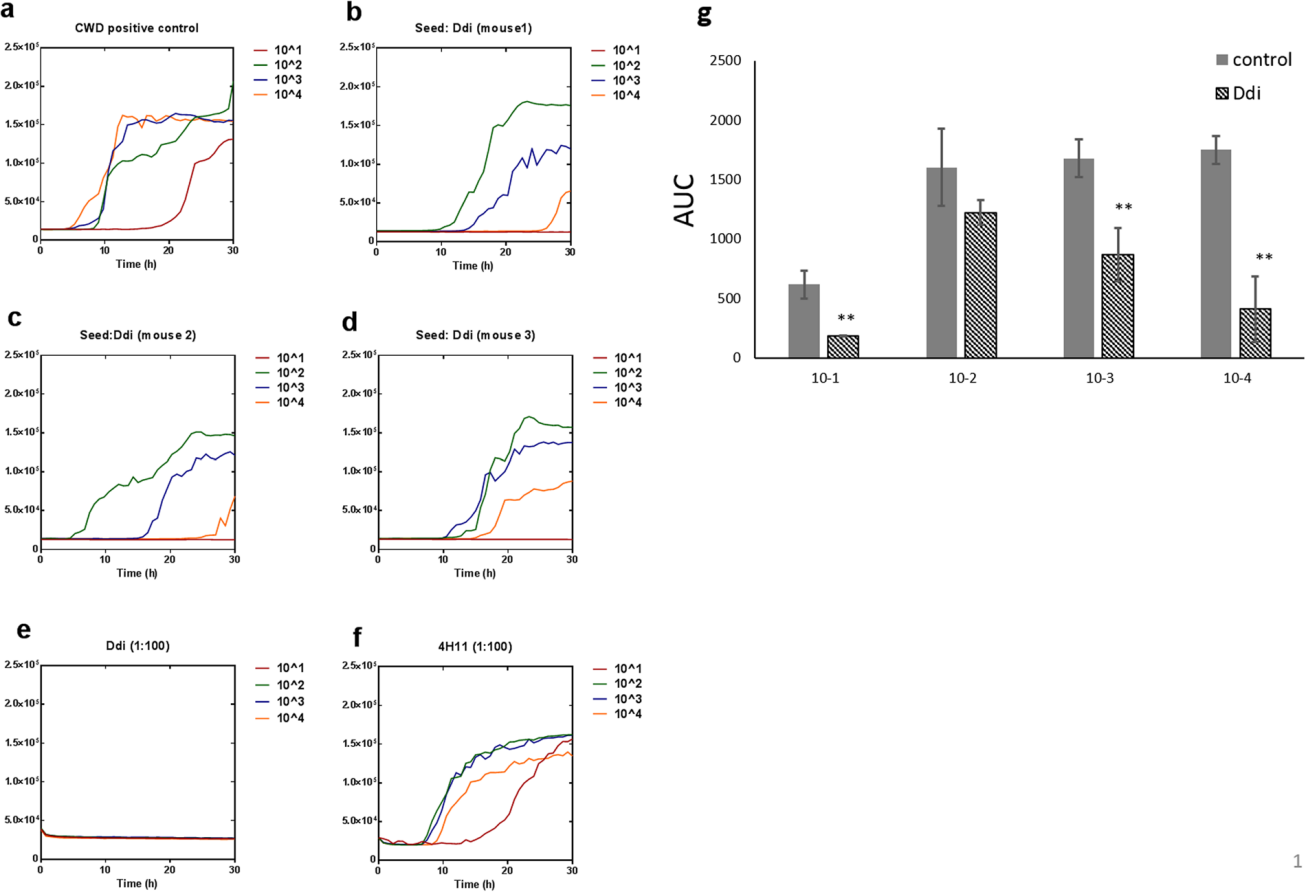
Pre-treatment of either recombinant PrP substrate or CWD seed with Ddi post-immune sera blocks CWD prion conversion in RT-QuIC assay. (**a**) CWD positive control for RT-QuIC in which mouse rPrP was used as substrate and 10% brain homogenate of a terminally ill Tg cerPrP (1536^+/+^) mouse infected with mule deer CWD was used as seed. (**b**–**d**) The same seed was incubated with post-immune sera (1:1) from 3 individual Ddi vaccinated mice for 1 hour at 37 °C before performing the RT-QuIC assay. (**e**) Mouse rPrP substrate was incubated for 30 min at 37 °C with post-immune sera of mice vaccinated with Ddi in dilution 1:100, before performing RT-QuIC. (**f**) Mouse rPrP substrate was incubated for 30 min at 37 °C with mAb 4H11, diluted 1:100, before performing RT-QuIC. Four tenfold dilutions of seed were used (10^−1^ to 10^−4^) and each curve represents the average of 4 technical replicates.Y axis represents the relative fluorescence units (RFU). (**g**) Area under the curves (AUC) for both positive control (**a**) and the Ddi-treated seed (**b**–**d**) were calculated for every dilution and represented as mean of three independent curves ± SD. **P < 0.01 is considered significant.

**Figure 5:**
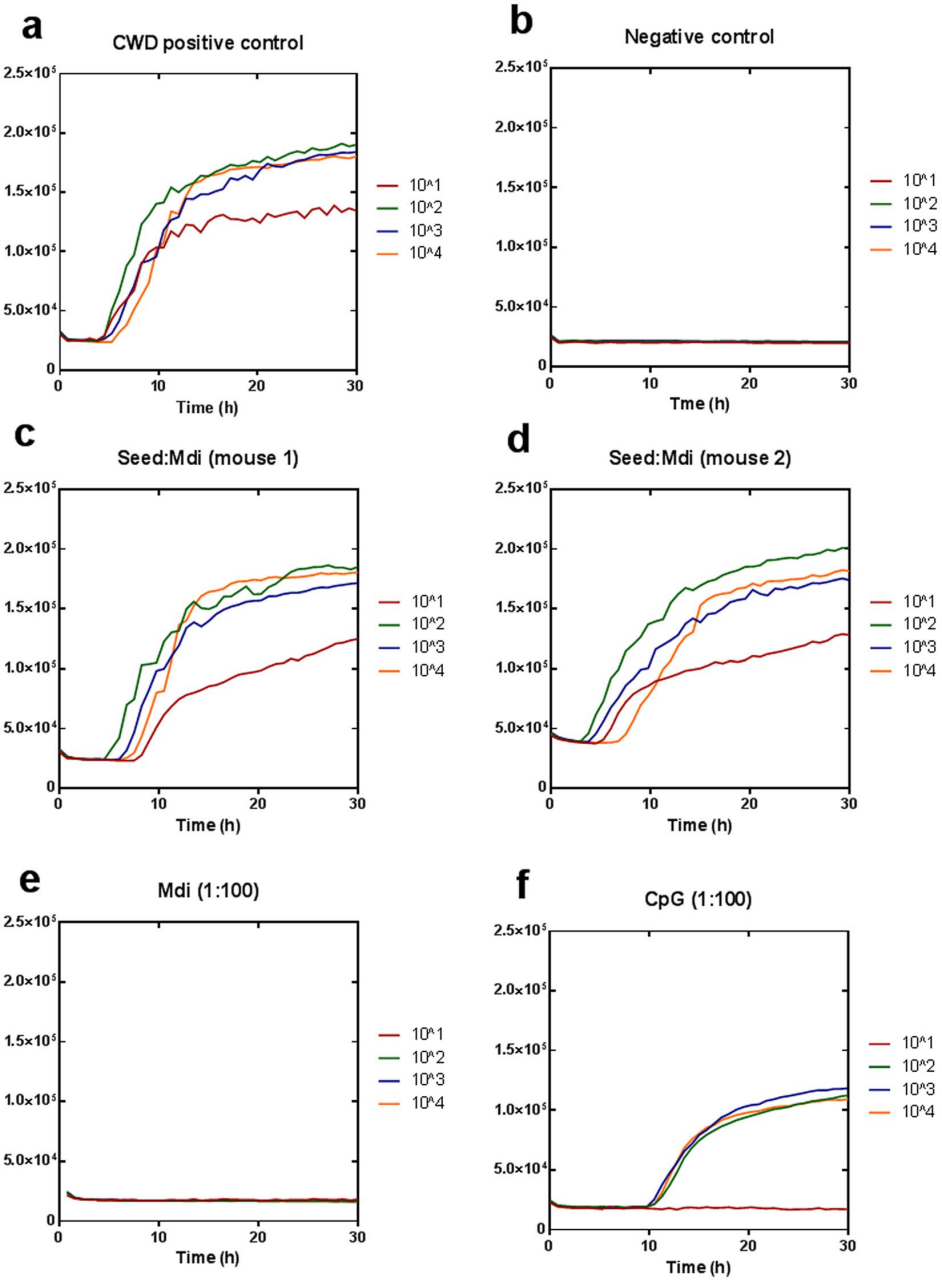
Pre-treatment of recombinant PrP substrate with Mdi post-immune sera blocks CWD prion conversion in RT-QuIC assay. (**a**) CWD positive control for RT-QuIC in which the substrate was mouse rPrP and the seed was 10% brain homogenate of a terminally ill Tg cerPrP (1536^+/+^) mouse infected with mule deer CWD. (**b**) Brain homogenate of an uninfected tg cerPrP (1536^+/+^) mouse was used as negative control. (**c**,**d**) The same CWD seed was incubated with post-immune sera (1:1) from 2 individual Mdi vaccinated mice for 1 hour at 37 °C before performing the RT-QuIC assay. (**e**,**f**) Mouse rPrP substrate was incubated for 30 min at 37 °C with post-immune sera of mice vaccinated with Mdi or adjuvant only (CpG) in dilution 1:100, before performing RT-QuIC. Four tenfold dilutions of seed were used (10^−1^ to 10^−4^) and each curve represents the average of 4 technical replicates. Y axis represents the relative fluorescence units (RFU).

To test the effect of self-antibodies on CWD prion conversion *in vitro*, we used real-time quacking-induced conversion (RT-QuIC) which is considered an ultra-sensitive state-of-the-art technique for detection of prions^24^. Recently, RT-QuIC has been also used for studying the anti-prion activity of several drugs and chemicals^25, 26^. This assay allows testing separately the binding of antibodies to PrP^C^ (substrate) or PrP^Sc^ (seed). Pre-incubation of the seed (10% brain homogenate of terminally ill mice infected with mule deer CWD) with Ddi post-immune sera (3 individual mice shown) decreased the conversion activity significantly in 3 dilutions (10^−1^, 10^−3^ and 10^−4^) (Fig. 4b,c,d and g), whereas Mdi post-immune sera pretreated seed showed seeding activity similar to the untreated positive control (Fig. 5a,c and d). This indicates that antibodies in Ddi post-immune sera have the ability to partially sequester infectious prion protein in the CWD seed and diminish its conversion activity. Notably, pre-incubation of substrate (mouse recombinant PrP) with either Mdi or Ddi post-immune sera in dilution 1:100 for 30 min totally blocked CWD propagation in RT-QuIC (Figs 4e and 5e). This blocking effect on CWD conversion activity decreased when the substrate was incubated with more diluted post-immune sera (1:250) (Fig. S2), with a decline in the RT-QuIC kinetics rather than full blocking.

**Figure 6:**
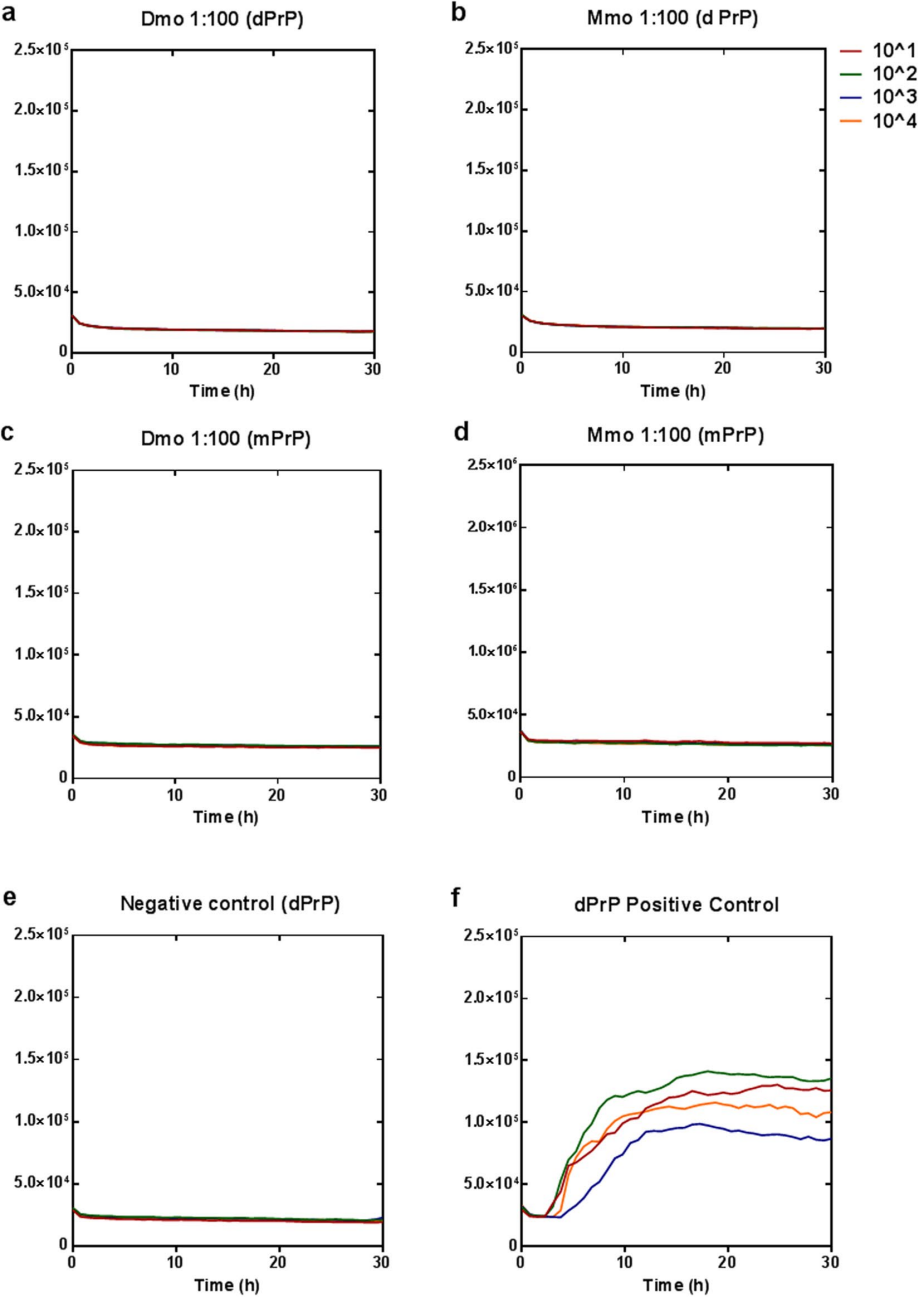
Pre-incubation of both mouse and deer PrP substrate with post-immune sera impedes CWD prion conversion in RT-QuIC. (**a**,**b**) Deer rPrP substrate was pretreated with post-immune sera of mice vaccinated with either Dmo (1:100) or Mmo (1:100) rPrPs, respectively, for 30 min at 37 °C before performing RT-QuIC. (**c**,**d**) Mouse rPrP was pretreated with post-immune sera of mice vaccinated with Dmo (1:100) or Mmo (1:100), respectively, for 30 min at 37 °C before performing RT-QuIC. (**e**) Brain homogenate of uninfected tg cerPrP (1536^+/+^) mice was used as negative control. (**f**) Positive control in which deer rPrP was used as a substrate. The brain homogenate of mule deer CWD infected tg cerPrP (1536^+/+^) mice was used as seed in all RT-QuIC experiments. Four 10-fold dilutions of seed were used and each curve represents the average of 4 technical replicates. Y axis represents the relative fluorescence units (RFU).

Importantly, incubation of substrate with post-immune sera from adjuvant-only (CpG) control mice only marginally reduced at dilution 1:100, but did not block CWD prion conversion in RT-QuIC (Fig. 5f). Vaccination with recombinant PrPs produced more efficient antibodies, which were able to totally block the conversion activity at the same dilution. Interestingly, self-antibodies in post-immune sera (either Mdi or Ddi) revealed a superior inhibitory effect on CWD conversion as compared to an anti-PrP mouse monoclonal antibody (mAb 4H11) at the same dilution (1:100) (Fig. 4f). This indicates more efficient crosslinking of PrP accomplished by the post-immune sera, effectively hampering conversion of the substrate. To test the influence of the recombinant PrP substrate (rPrP) on the inhibitory activity of post-immune sera, we decided to use both deer and mouse rPrP substrates. Treatment of either mouse or deer rPrP with post-immune antibodies of mice vaccinated with mouse (Mmo) or deer monomer (Dmo) blocked CWD conversion in RT-QuIC (Fig. 6). Again, the total inhibitory effect achieved by the post-immune sera on the CWD conversion at dilution 1:100 was greater than the partial inhibition achieved by the same dilution of monoclonal antibody 4H11 (Fig. 4f).

Taken together, these data demonstrate that the induced self-antibodies in post-immune sera strongly bind to recombinant PrP and make it ineligible for CWD prion conversion in RT-QuIC reaction. Moreover, Ddi post-immune sera also bind to CWD prions in the RT-QuIC seed and decrease their conversion activity.

### Self-antibodies reduce prion propagation in ScN2a and CWD-infected RK13 cells

**Figure 7:**
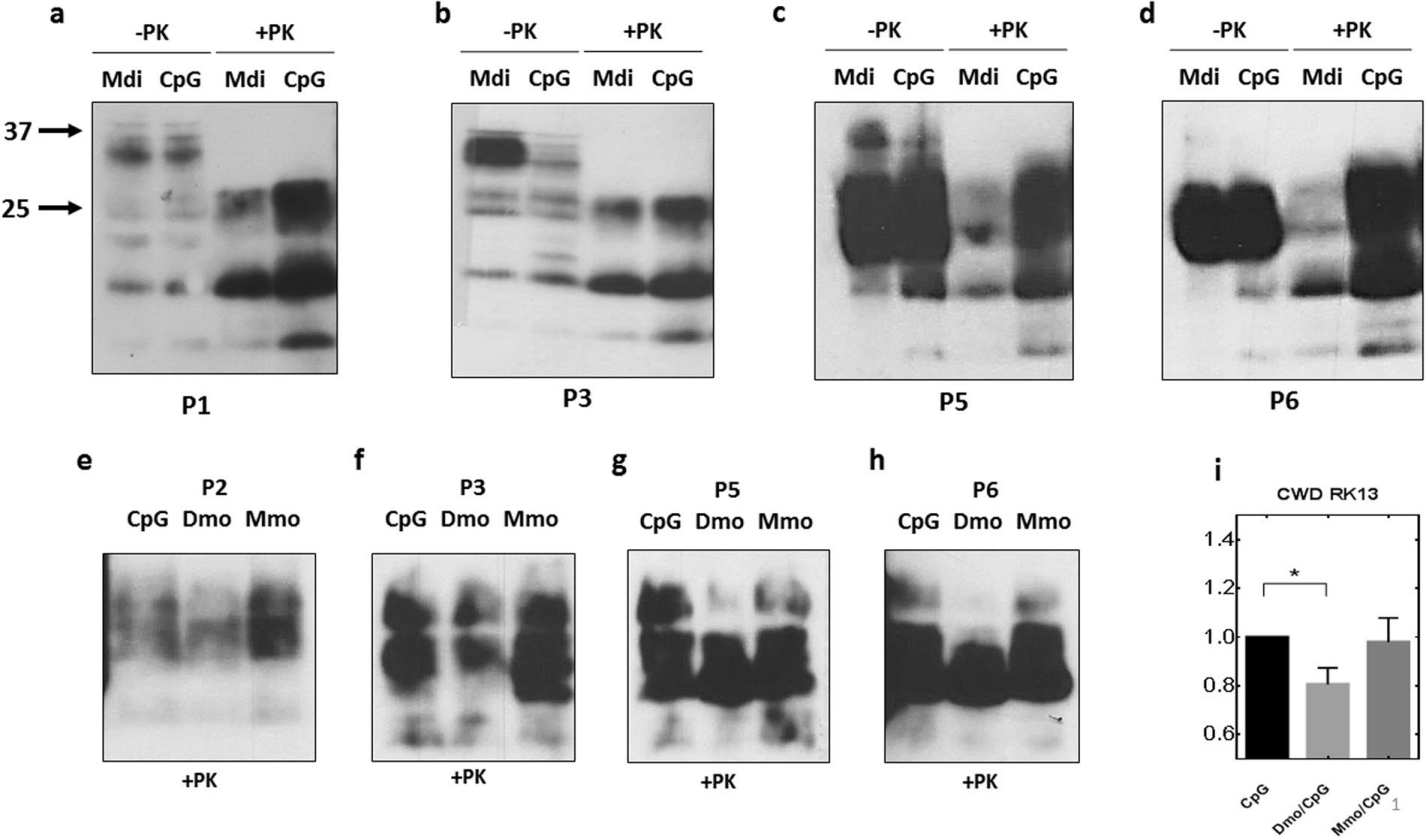
Post-immune sera interfere with PrP^Sc^ biogenesis in both ScN2a and CWD-infected RK13 cells. (**a**–**d**) ScN2a cells were treated with post-immune sera from mice vaccinated with Mdi rPrP or adjuvant alone (CpG) while passaging the cells every 5 days for 6 passages. Sera were used in dilution 1:100 in culture media. Cells from every passage were lysed and subjected to PK digestion, or not (+PK, −PK). Lysates were immunoblotted with either pAb 142 (**a**,**b**) or mAb 4H11 (**c**,**d**,**e**–**h**) CWD-infected RK13 cells expressing cervid PrP^C^ (CWD RK13) were treated with post-immune sera from mice vaccinated with Mmo rPrP, Dmo rPrP, or adjuvant alone (CpG), while passaging the cells every 7 days for 6 passages. Sera were used in dilution 1:100 in culture media. Cells from every passage were lysed and subjected to PK digestion (+PK). The lysates (all + PK) were immunoblotted with mAb 4H11. (**i**) Densitometric analysis for the CWD RK13 cell immunoblots. Data are represented as percentage of control (CpG). *P < 0.05 is considered significant.

The N2a neuroblastoma cell line persistently infected with 22 L prions (ScN2a) is widely used as a cellular model for prion diseases^27^. To test the efficiency of self-antibodies in restraining prion propagation, we treated ScN2a cells with post-immune sera of Mdi or CpG-only vaccinated mice. Cells treated with Mdi post-immune sera revealed markedly reduced PrP^Sc^ levels in all passages compared with the CpG-only sera treated cells, as shown by proteinase K resistant (+PK) signals (Fig. 7a–d). Treatment of persistently CWD-infected RK13 cells (over-expressing cervid PrP) with post-immune antibodies from Dmo or Mmo vaccinated mice resulted in significantly reduced PrP^Sc^ amounts only in cells treated with Dmo post-vaccination sera (Fig. 7e–i). This indicates that the autoantibodies formed against deer PrP (Dmo) are more efficient than the cross-reactive antibodies (Mmo) in suppression of CWD prion propagation. Unfortunately, we were not in the position to test also Ddi and Mdi post-immune sera in CWD prion-infected cells due to lack of sera.

In summary, these data show that induced self-antibodies have the potential to interfere in cellular prion propagation.

### Autoantibodies produced in Ddi and Dmo vaccinated mice are directed against similar linear epitopes

**Figure 8:**
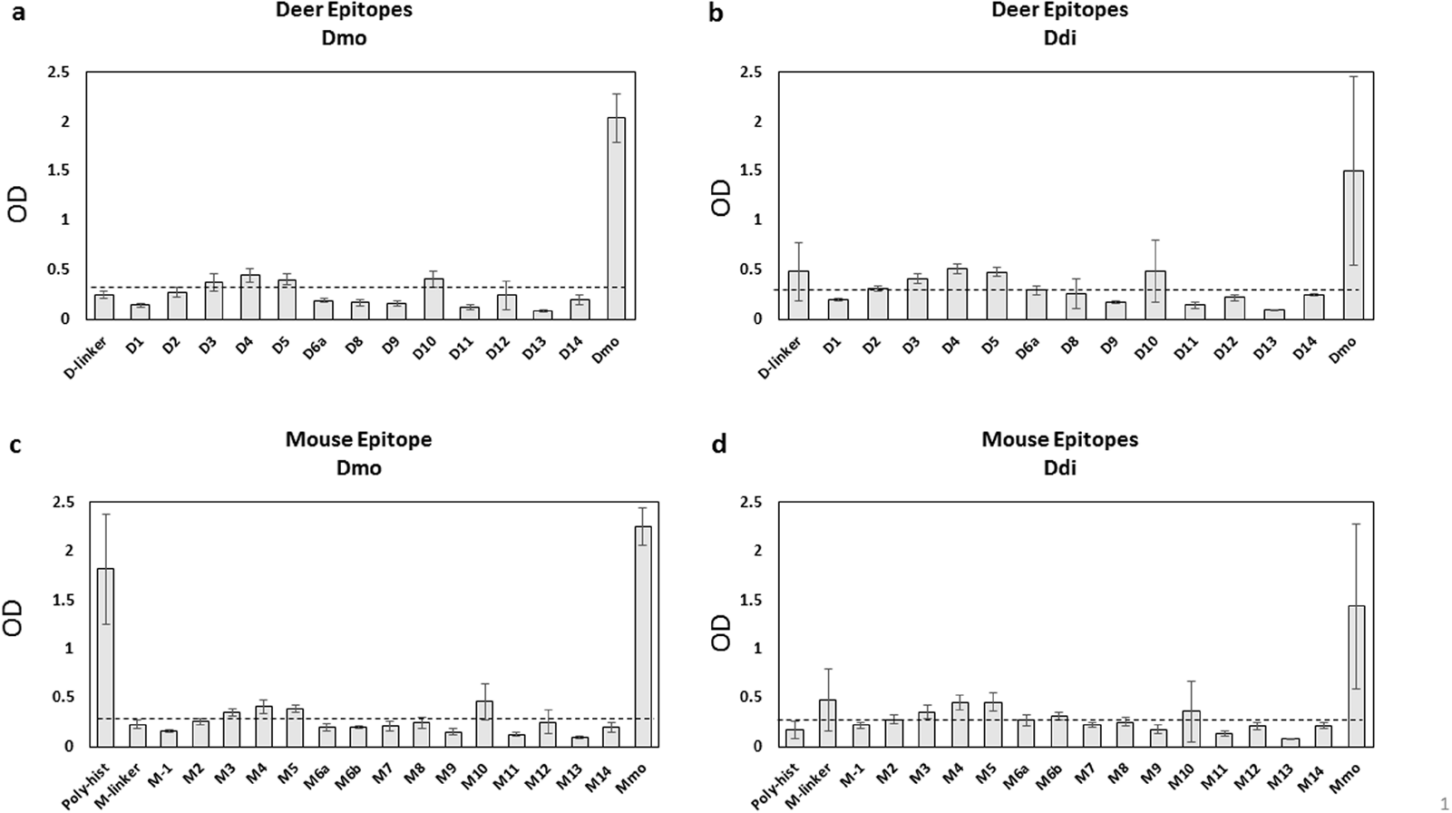
Linear epitope mapping for Dmo and Ddi post-immune sera. The optical density (OD) at 405 nm indicates the reactivity of sera from either Dmo (**a**,**c**) or Ddi (**b**,**d**) immunized mice against linear epitopes of deer (**a**,**b**) or mouse PrP (**c**,**d**) in ELISA. The dashed horizontal line represents the cut-off, which is 3 times the average of pre-immune sera. Data are presented as mean ± SD of results from three individual mice of each group. The amino acid sequences for linear epitopes is shown in Supplementary Tables 1 and 2.

We used an overlapping polypeptide library representing the mature full-length version of both deer and mouse PrP for performing a linear epitope mapping of antibodies in Ddi and Dmo post-vaccination sera. This also should show differences in reactivity to deer (“self”) vs. mouse epitopes (“non-self”). Since we used sera from three individual mice, this reflects the average reactivity in a group. As shown in Fig. 8c, there was substantial reactivity of Dmo sera against the poly-histidine tag which is entirely absent in Ddi post-immune sera. Other than the poly-histidine epitope, we could not detect significant differences between Dmo and Ddi post-immune sera in terms of reactivity to linear epitopes, nor was there a difference between mouse and deer epitopes (Fig. 8a–d). Of note, when testing sera from individual mice we observed some variability for reactivity against epitopes 8 and 10 (*data not shown*). Since this assay only detects reactivity against linear epitopes, we cannot exclude a different reactivity against discontinuous and conformational epitopes. This will need further investigations with newly immunized mice in order to generate the amounts of sera as needed for such studies.

Taken together, our linear epitope mapping did not provide major differences between Dmo and Ddi sera, nor was there a strong difference in reactivity between mouse and deer epitopes.

## Discussion

The management of chronic wasting disease is extremely challenging because of the very effective lateral transmission, the environmental involvement, and the existence in free ranging cervid populations^28, 29^. Active vaccination would be an ideal approach for containment of CWD. The major obstacle with using immune system mediated strategies is that prion diseases do not intrinsically elicit immune reactions. In prion propagation, the normal cellular prion protein is post-translationally converted into the pathologic isoform PrP^Sc^. Both PrP^C^ and newly generated PrP^Sc^ are considered self proteins by the host immune system, as they share the same primary structure. Prion infections do therefore not induce any humoral or cellular immune responses, as the components of the adaptive immune system deal mainly with linear epitopes^30, 31^.

On the other hand, there is solid experimental evidence that experimental immunization strategies, either passive or active, are remarkably effective against prion infection *in vitro* in prion-infected cells and *in vivo* in mouse models^8–10^.

The challenge is to overcome self-tolerance in vaccination approaches in a way which is protective without inducing side effects.

Our previous work on vaccination in wild-type mice revealed that aggregation-prone dimeric recombinant PrP, which has a unique conformation intermediate between PrP^C^ and PrP^Sc^, has the potential to overcome self-tolerance and provide protection against prion infection *in vitro* and *in vivo* ^10, 11, 17^. In the current study, we investigated immunogenicity of dimeric cervid (self) and murine (non-self) PrP and the protective effects of induced antibodies on CWD propagation *in vitro*. Mouse and cervid PrP differ by around 20% on the amino acid level (Fig. S1).

Self-tolerance is a major obstacle in eliciting anti-PrP immune responses. Using the appropriate adjuvant is crucial to break the self-tolerance. Our results revealed that deoxycytidyl-deoxyguanosine (CpG) improved the humoral response against dimeric deer PrP (Ddi). Our finding is in line with a previous study which reported that CpG stimulated the B cell repertoire against PrP immunogens^32^. Notably, no overt side effects have been observed in vaccinated mice, both in the cervid PrP-expressing transgenic mice used here, or in wild-type mice used before^17^. This was not unexpected, as various studies had revealed that PrP can be knocked out in mice without inducing any obvious phenotype^33, 34^.

As expected, non-self immunogens (Mmo, Mdi) produced higher humoral immune responses compared with self immunogens (Dmo, Ddi) when tested in ELISA. Yet, only the antibodies from deer dimer (Ddi)-vaccinated mice were able to detect CWD PrP^Sc^ in immunoblots when used as primary antibodies. Compared with mouse dimer (Mdi), Ddi post-immune sera showed also more inhibition of RT-QuIC reaction when pre-incubated with the CWD seed. Given that the antibody titer of Mdi-induced sera was much higher than that of the Ddi-induced ones, this suggests that antibody titers are likely not correlated with anti-prion protection efficacy. It also indicates that self-antibodies are superior in detecting PrP^Sc^ and PrP^Sc^-specific epitopes. However, the latter possibility has to be tested by performing a conformational epitope mapping. Our linear epitope mapping did not show considerable differences when averaging results from individual mouse sera, although some individual mouse sera indicated more reactivity in epitopes 8 and 10 (Fig. 8 and data not shown).

As shown by the immunoblots in our study, only Ddi post-vaccination sera were able to detect both PrP^C^ and PrP^Sc^ in mule deer and elk brain homogenates when used as primary antibodies. Given that we used the same dilution for all post-immune antibodies and that Ddi-sera had the lowest ELISA titer, dimeric deer PrP (Ddi) must have induced more efficient antibodies than monomeric deer PrP (Dmo). This may be explained by the difference in the conformation of these immunogens. As shown by the epitope mapping (Fig. 8c), there was a substantial response against the polyhistidine tag only in Dmo-induced sera, indicating a different folding of Ddi and Dmo immunogens. Accordingly, the high ELISA titers of Dmo post-immune sera are not reflected by high reactivity against cervid PrP^C^ in other test formats.

There is a consensus in the prion field that preceding cell surface expression of PrP^C^ is a pivotal requisite for cellular prion biogenesis^35, 36^. Consequently, antibodies which can bind to native and surface-located PrP^C^ will have the potential to interfere in prion pathogenesis^9–11^. Interestingly, the post-immune antibodies in our study were able to bind to surface-located PrP^C^ as shown by confocal microscopy and solubility assay. This very likely explains the protective effect of post-immune sera against prion propagation in ScN2a and ScRK13 cells. Post-immune antibodies were able to bind, cross-link and sequester PrP^C^, forming a shield which inhibits the contact between PrP^C^ substrate and PrP^Sc^ template needed in cellular prion conversion.

RT-QuIC assay has been used widely as a diagnostic technique for ultra-sensitive detection of prions as evidenced by prion-induced conversion activity, not only in brain but also in cerebrospinal fluid, urine, feces and saliva^37–40^. Recently, the RT-QuIC assay has emerged as a promising platform for screening of chemical compounds inhibiting the prion conversion process^25, 26^. There are no reports yet on screening the efficiency of anti-PrP antibodies for inhibiting prion conversion.

As our prion challenge studies in vaccinated mice are still ongoing, we looked for a screening test that mimics CWD propagation in cervids. To the best of our knowledge, our study is the first to use the RT-QuIC assay as screening platform for testing the efficiency of anti-PrP auto-antibodies in inhibiting prion propagation. In RT-QuIC, we used CWD brain homogenate as a seed to mimic CWD propagation in cervids. The post-vaccination sera from Ddi-immunized mice were the only sera able to mitigate CWD prion conversion activity when incubated with the seed (Fig. 4). This is accordant with the ability of the Ddi post-immune sera to detect PrP^Sc^ from deer and elk in immunoblots. However, post-immune sera from all vaccinated groups were able to interfere with CWD prion conversion when incubated with the PrP substrate, indicating that post-immune antibodies from all groups have the ability to bind and cross-link the recombinant PrP used as RT-QuIC substrate.

Interestingly, the inhibition of seeding activity achieved by post-immune self-antibodies was greater than that of mAb 4H11 at the same dilution (1:100), suggesting efficient cross-linking of the substrate by the polyclonal antibodies. This efficient binding and cross-linking may cause steric hindrance that inhibits the contact between PrP^C^ and PrP^Sc^. Interestingly, CpG post-immune sera decreased the conversion activity of CWD prions in RT-QuIC assay only very marginally, much less than the blocking effect caused by Mdi and Ddi post-immune sera at the identical dilution. The subtle effect of control sera may be explained, at least partially, by the existence of some autoantibodies against PrP as reported by previous studies^41, 42^. Whether using deer or mouse substrate did not affect the inhibitory effect of post-immune sera (Fig. 6), suggesting a cross-reactivity of antibodies against both recombinant cervid and murine PrP. Although the RT-QuIC assay cannot replace the animal experiments, it provides a suitable *in vitro* screening tool for the prophylactic efficiency of anti-PrP antibodies.

In conclusion, our study demonstrates that the multimeric cervid recombinant PrP (Ddi) can efficiently overcome the self-tolerance to PrP and induce self-antibodies in transgenic mice expressing deer PrP, representing an immunological self-situation. Auto-antibodies elicited by Ddi immunogen can efficiently bind to surface-located authentic PrP^C^ on cervid PrP expressing cells, interfere in prion propagation in CWD-infected cells, and detect CWD PrP^Sc^ in immunoblot. Moreover, induced post-immune self-antibodies inhibited the CWD seeding activity in RT-QuIC, which can be used as an *in vitro* screening platform for testing the potential of anti-PrP antibodies to inhibit CWD propagation.

## Materials and Methods

### Ethics statement

All animal experiments were conducted strictly following the guidelines of the Canadian Council for Animal Care (CCAC) and were approved by the institutional Health Sciences Animal Care Committee (HSACC) (protocol: AC14-0122).

### Mice

The transgenic mice, tg (cerPrP) 1536^+/+^ used in this study were kindly provided by Dr. Glenn Telling (Colorado State University, Colorado, USA). The generation, genotyping and susceptibility of these mice were described previously^22^. Only the female mice in homozygous background were used in this study.

### Immunization

Female tg(cerPrP)1536^+/+^ mice were vaccinated by injecting 100 µg protein/mouse subcutaneously as a priming dose followed by four booster injections (50 µg) at three weeks’ intervals. Either, alum 1:1 (v:v) (Imjet, Thermosientific) or CpG (5 µM) (InvivoGen) were used as adjuvants. The blood samples were drawn before injection (pre-immune sera) and 10 days after the last boost (post-immune sera).

### Preparation and purification of immunogens

Construction of monomeric and dimeric mouse PrP (Mmo and Mdi) was done as described previously^10^. In brief, the murine PrP gene (prnp-A) encoding the monomeric PrP (Mmo; aa 23–231) was subcloned into bacterial expression vectors. Mdi consists of two murine PrP moieties (aa 23–231), each tagged with 3F4 and covalently linked by a 7-aa linker (AGAIGGA). The mule deer gene constructs encoding monomeric (Dmo) or dimeric PrP (Ddi) were synthesized by GeneArt. All constructs were cloned into the bacterial expression vector pQE30 (Qiagen). Expression of immunogens was performed in *Escherichia coli* strain BL21-Gold(DE3) pLysS (Stratagene) as previously described^10, 17^. Briefly, the bacterial pellet was lysed with lysis buffer pH 7.8 (6 M guanidinium hydrochloride [GdnHCl], 20 mM sodium-phosphate, 500 mM NaCl). After centrifugation at 6000 × g for 30 min, supernatant was transferred to Ni-NTA Superflow resin beads (Quiagen) and incubated at 4 °C for 1 h, followed by two washing steps with binding buffer pH 7.8. Then, the resin-bound protein was washed 6 times with washing buffer pH 6.3. Resin was transferred to the columns and the protein was eluted with elution-buffer pH 6.3 and refolded by dialysis against 10 mM NaAc buffer (pH 4.5) overnight at 4 °C (10,000 MWCO Dialysis Cassettes; Pierce). Protein concentration was measured with BCA kit (Pierce, Thermo scientific).

### Enzyme linked immunosorbent assay (ELISA)

High-binding 96 well plates (Greiner Bio-One GmbH) were coated with 1 μg of recombinant PrP in sodium-carbonate buffer (pH 9.5) and incubated overnight at room temperature (RT). After washing with PBST, the plates were blocked for 2 h with 3% BSA, at 37 °C. Sera of mice were diluted in 3% BSA and then incubated for 1 h at 37 °C. After washing, plates were incubated with HRP-labeled anti-mouse IgG antibody (Jackson Immuno-research Lab) for 1 h at 37 °C. The plates were washed again and incubated with ABTS (2,2′-Azino-bis(3-Ethylbenzthiazoline-6-Sulfonic Acid) peroxidase substrate system (KPL). After 10 minutes at 25 °C, the optical density at 405 nm (OD405) was measured using BioTek Synergy HT. The cut-off was defined as three times the OD405 value of 1:100 diluted pre-immune sera.

### Maintenance of cell culture

The mouse neuroblastoma cell line N2a was initially obtained from ATCC (CCL-131) and cultured as described previously^43^. RK13 cells expressing cervid PrP (cerRK13) were a generous gift from Dr. Glenn Telling (Colorado State University, Colorado, USA). Cells were cultured in DMEM, 1% penicillin/streptomycin, 10% FBS, 1 μg /ml puromycin, and 200 μg/ml G418. The cells were infected with 1% brain homogenate of a terminally ill tg (cerPrP) 1536^+/+^ mouse infected with white-tailed deer CWD to get the ScRK13 cells^44^.

### Solubility assay

Solubility assays were performed as described previously^27^. Briefly, post-nuclear lysates were ultracentrifuged at 100,000 × g for 1 hour at 4 °C (Beckmann TL 100 ultracentrifuge; rotor TLA-45) after adding N-lauryl- sarcosine (1%). Pellet fractions containing insoluble PrP were resuspended in 20 μl TNE buffer and 10 μl sample buffer.

### Proteinase K (PK) digestion and Western blotting

Immunoblot analysis was performed as previously described^27^. Briefly, confluent cells were lysed in lysis buffer. The lysates were incubated with 20 µg/ml PK for 30 min at 37 °C, the digestion was stopped with 0.5 mM Pefabloc and methanol precipitation was done. For non-PK digested samples (-PK), Pefabloc was added directly and precipitated with methanol. Pellets were re-suspended in TNE buffer. For brain homogenates, 50 µg/ml of PK was used for 1 h at 37 °C, followed by adding the sample buffer. Samples were run on 12.5% SDS-PAGE, electro-blotted on Amersham Hybond P 0.45 PVDF.

### Immunofluorescence

Uninfected N2a or cerRK13 cells reaching 80% confluency were chilled in ice-cold extracellular solution (ECS): 150 NaCl, 5 KCl, 2.5 CaCl^2^, 1 MgCl^2^, 10 HEPES, 10 D-Glucose (mM) and 0.5 µM CuSO^4^ for a 5 min incubation at 4 °C and then incubated with different antibodies or sera dilutions in the same ECS buffer for 30 min at 4 °C. For N2a cells, dilutions were mAb 4H11 at 1:200, and Mdi at 1:100. For cerRK13 cells, mAb 4H11 was at 1:400, Ddi at 1:50, and CpG was at 1:100 dilution. Cells were then rinsed in ice-cold ECS three times and either fixed in 4% paraformaldehyde for 30 min at RT (for 4 °C surface binding) or returned to 37 °C incubator for additional 60 min incubation in cell culture medium and then fixed (for 60 min internalization). Alexa fluor 488 goat anti-mouse secondary antibodies (Jackson Immuno-Research lab) were 1:200 diluted in PBS containing 5% fetal bovine serum (FBS) to visualize surface binding at 4 °C under non-permeabilized conditions, or in PBS containing 5% FBS and 0.5% Triton X- 100 to reveal internalization of antibodies under permeabilized condition, respectively.

### Epitope mapping

For epitope mapping, we used a polypeptide library synthesized by Peptide 2.0 (Chantilly, VA, USA). This library consists of 20-aa peptides with 5 aa overlap, encompassing full-length mature cervid and murine PrPs as depicted in Supplementary Table 1 and 2. Peptide 6a incorporates the 3F4 epitope, peptide 6b represents the corresponding murine sequence. CovaLink NH microtiter plates were activated with disuccinimidyl suberate (DSS) (Thermo Scientific, USA) bifunctional linker in carbonate buffer and incubated overnight at RT with peptides (10 µg/well) or recombinant protein (1 µg/well) as control. The coated plates were blocked with BSA and incubated with pre-diluted sera (1:100), washed and incubated with secondary antibodies. After washing and incubation with ABTS substrate, the optical density was measured at 405 nm.

### Real Time Quacking Inducing Conversion (RT-QuIC)

#### Preparation of recombinant protein

Preparation of recombinant prion proteins was performed as described previously^45^. Briefly, mouse PrP (aa 23–231) was cloned into pET-41 plasmids, transformed into *E*. *coli* Rosetta, and bacteria cultured in LB media supplemented with kanamycin (0.05 mg/ml) and chloramphenicol (0.034 mg/ml). The Overnight Express Autoinduction System (Novagen) was used to induce protein expression. Inclusion bodies were isolated from pelleted cells using Bug Buster Master Mix (Novagen) and stored at −20 °C. For purification of recombinant PrP, inclusion bodies were solubilized in (8 M guanidine-HCl, 100 mM Na-phosphate, 10 mM Tris-HCl, pH 8.0) and incubated on the rocker for 1 h at RT. Ni-NTA Superflow resin beads (Quiagen) were incubated in denaturing buffer for 1 h at RT. Solubilized inclusion bodies were centrifuged at 16,000 × g for 5 min, the supernatant added to the beads and incubated for 1 h with gentle rocking. Beads were then packed into a XK16 glass column. Using an Amersham ÄKTA Explorer FPLC unit running with Unicorn software (5 version, GE Healthcare Life Sciences), protein was refolded by a gradient from 100% denaturing buffer to 100% refolding buffer over 4 h. The column was washed for 30 min with refolding buffer and proteins eluted using a linear gradient from 100% refolding buffer to 100% elution buffer. The central portions of the A280 UV peak were collected into dialysis buffer and purified protein was dialyzed in dialysis buffer overnight at 4 °C. The protein solution was filtered with a prewashed 0.22 µm filter, concentration was measured using BCA assay (Thermo-Scientific) and stored at −80 °C.

#### RT-QuIC assay

RT-QuIC was performed as described^39^. Briefly, reactions were set up in assay buffer containing 20 mM Na-phosphate, pH7.4, 300 mM NaCl, 1 mM EDTA, 10 μM Thioflavin T and 0.1 mg/ml rPrP substrate. 98 μl aliquots were added to the wells of a black-walled 96-well optical bottom plate (Nalge Nunc International, Nunc). Tenfold serial dilutions of brain homogenate were prepared in 0.5 ml microtubes with seed buffer containing 0.02% SDS. Quadruplicate reactions were seeded with 2 µl of each dilution for a final reaction volume of 100 µl. Plates were sealed with Nunc Amplification Tape (Nalge Nunc International) and incubated in a FLUOstar Omega plate reader for 30 h at 42 °C, with cycles of 60 s shaking and 60 s rest throughout the incubation. ThT fluorescence was measured (450 nm excitation and 480 nm emission) every 15 min. RT-QuIC data were averaged from four replicates which was plotted against reaction time. Samples were scored positive if at least 50% of replicates reached a ThT fluorescence cut-off.

### Statistical analysis

GraphPad Prism version 6.01 was used for performing the statistical analysis and plotting the graphs. The area under the curve (AUC) was calculated using Excel. Statistical significance was tested using unpaired student t test for the AUC and using ANOVA for CWD-RK13 immunoblots densitometry, and represented as ns: not significant, *p < 0.05, **p < 0.01.

## Electronic supplementary material

supplementary info 1

Supplementary Information
