# Supplementary material for: Immunization of cervidized transgenic mice with multimeric deer prion protein induces self-antibodies that antagonize chronic wasting disease infectivity in vitro
Source: Sci Rep. 2017 Sep 5;7:10538. doi: 10.1038/s41598-017-11235-8 (PMC5585258; doi:10.1038/s41598-017-11235-8)
Supplement: Supplementary file 1 — supplementary info 1 [file 41598_2017_11235_MOESM1_ESM.pdf]

Immunization of cervidized transgenic mice with multimeric deer prion protein induces self-antibodies that antagonize chronic wasting disease infectivity in vitro.

Dalia. H. Abdelaziz, Simrika Thapa, Basant Abdulrahman, Li Lu, Shikha Jain and Hermann M. Schatzl

Supplementary Data

Fig. S1

Supplementary figure 1: Alignment of amino acid sequence of mature prion protein of mule deer and mouse.

```
Mule deer PrP: KKRPKPGGGWNTGGSRYPGQGSPGGNRYPPQGGGGWGQPHGGGWGQPHGGGWGQPHGGGWGQPHGGGGWG
Mouse PrP:     KKRPKPGG-WNTGGSRYPGQGSPGGNRYPPQGGT-WGQPHGGGWGQPHGGSWGQPHGGSWGQPHGGG-WG

Mule deer PrP: QGG-THSQWNKPSKPKTNMKHVAGAAAAGAVVGGLGGYMLGSAMSRPLIHFGNDYEDRYYRENMYRYPNQ
Mouse PrP:     QGGGTHNQWNKPSKPKTNLKHVAGAAAAGAVVGGLGGYMLGSAMSRPMIHFGNDWEDRYYRENMYRYPNQ

Mule deer PrP: VYYRPVDQYNNQNTFVHDCVNITVKQHTVTTTTKGENFTETDIKMMERVVEQMCITQYQRESQAYYQRGAS
Mouse PrP:     VYYRPVDQYSNQNNFVHDCVNITIKQHTVTTTTKGENFTETDVKMMERVVEQMCVTQYQKESQAYYDGRRS
```

Fig. S2

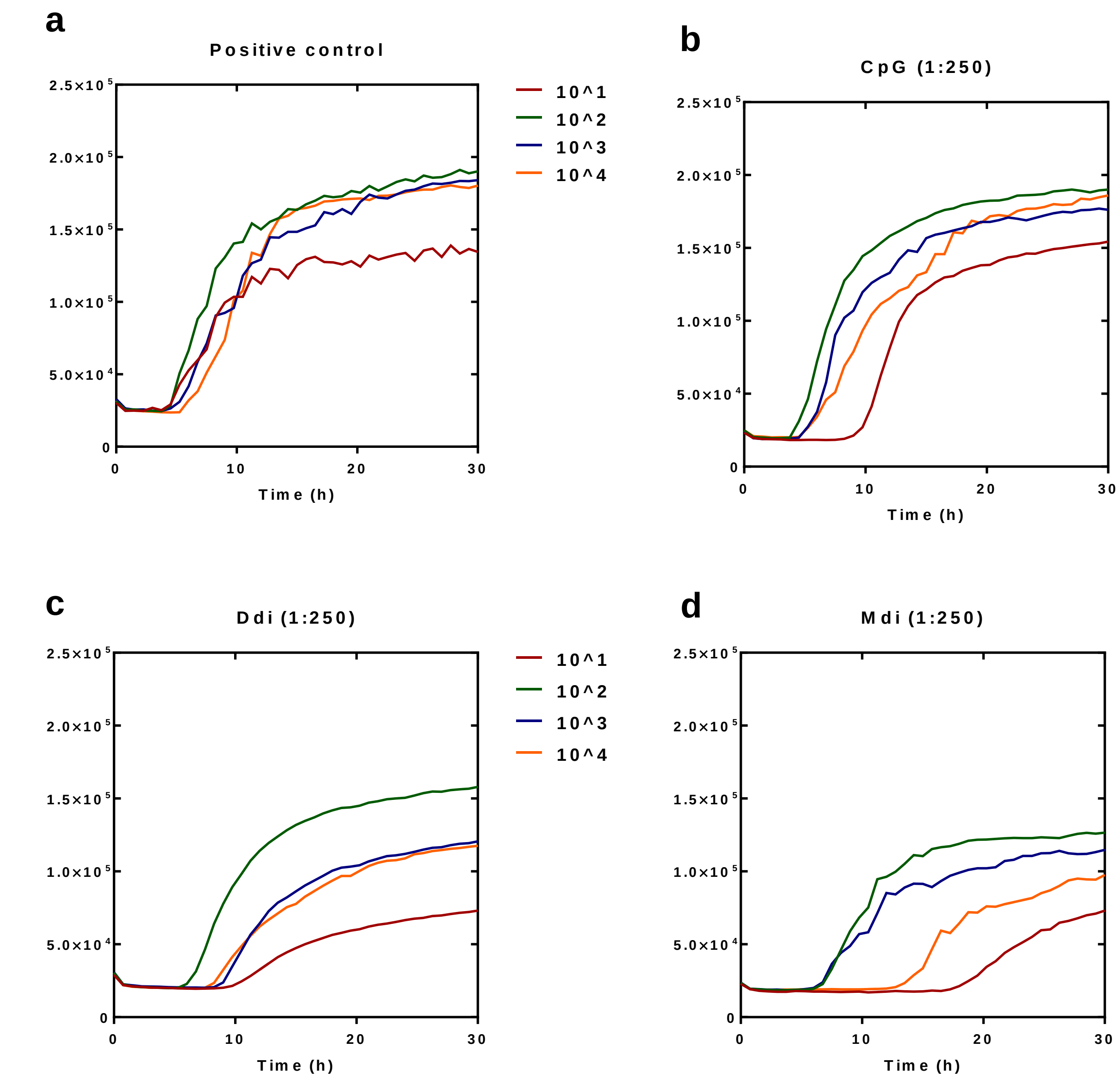

Supplementary figure 2: Pre-treatment of recombinant PrP with either Ddi or Mdi post-immune sera in dilution 1:250 decreases, but cannot block CWD prion conversion in RT-QuIC assay.

(a) CWD positive control for RT-QuIC in which the substrate was mouse rPrP and the seed 10% brain homogenate of terminally ill Tg cerPrP ($1536^{+/+}$) mice infected with mule deer CWD.

(b-d) Mouse rPrP substrate was incubated for 30 min at 37°C with post-immune sera of CpG, Ddi and Mdi immunized mice, respectively, in dilution 1:250, before performing RT-QuIC assay. Four tenfold dilutions of seed were used (10^{-1} to 10^{-4}) and each curve represents the average of technical quadruplicates.

Supplementary table 1: Mouse PrP Polypeptides library

Epitope	Amino acid Sequence
The poly-histidine	MRG SHH HHH HGS CKK RPK PG
M. linker region	DGR RSS AGA IGG AKK RPK P
M.1	KKRPKPGGWNTGGSRYPGQG
M.2	YPGQGSPGGNRYPPQGGT-WG
M.3	GGTWGQPHGGGWGQPHGGSW
M.4	HGGSWGQPHGGSWGQPHGGG
M.5	PHGGGWGQGGGTHNQWNKPS
M.6a	WNKPSKPKTNLKHVAGAAAA
M6b	WNKPSKPKTNMKHMAGAAAA
M.7	GAAAAGAVVGGLGGYMLGSA
M.8	MLGSAMSRPMIHFGNDWEDR
M.9	DWEDRYYRENMYRYPNQVYY
M.10	NQVYYRPVDQYSNQNNFVHD
M.11	NFVHDCVNITIKQHTVTTTT
M.12	VTTTTKGENFTETDVKMMER
M.13	KMMERVVEQMCVTQYQKESQ
M.14	QKESQAYYDGRRSS

Supplementary table 2: **Deer PrP Polypeptides library**

Epitope	Sequence
D. linker region	QRGASAGA IGG AKK RPK P
D.1	KKRPKPGGGWNTGGSRYPGQG
D.2	YPGQGSPGGNRYPPQGGGGWG
D.3	GGGGWGQPHGGGWGQPHGGGW
D.4	HGGGWGQPHGGGWGQPHGGG
D.5	PHGGGGWGQGGTHSQWNKPS
D.6a	WNKPSKPKTNMKHVAGAAAA
D.6b	WNKPSKPKTNMKHMAGAAAA
D.7	GAAAAGAVVGGLGGYMLGSA
D.8	MLGSAMSRPLIHFGNDYEDR
D.9	DYEDRYYRENMYRYPNQVYY
D.10	NQVYYRPVDQYNNQNTFVHD
D.11	NTFVHDCVNITVKQHTVTTTT
D.12	VTTTTKGENFTETDIKMMER
D.13	KMMERVVEQMCITQYQRESQ
D.14	QRESQAYYQRGAS
