# Supplementary material for: Immunization of cervidized transgenic mice with multimeric deer prion protein induces self-antibodies that antagonize chronic wasting disease infectivity in vitro
Source: Sci Rep. 2017 Sep 5;7:10538. doi: 10.1038/s41598-017-11235-8 (PMC5585258; doi:10.1038/s41598-017-11235-8)

Immunization of cervidized transgenic mice with multimeric deer prion protein induces self-antibodies that antagonize chronic wasting disease infectivity in vitro.

Dalia. H. Abdelaziz, Simrika Thapa, Basant Abdulrahman, Li Lu, Shikha Jain and Hermann M. Schatzl

Supplementary information

Original blots

Figure 1c

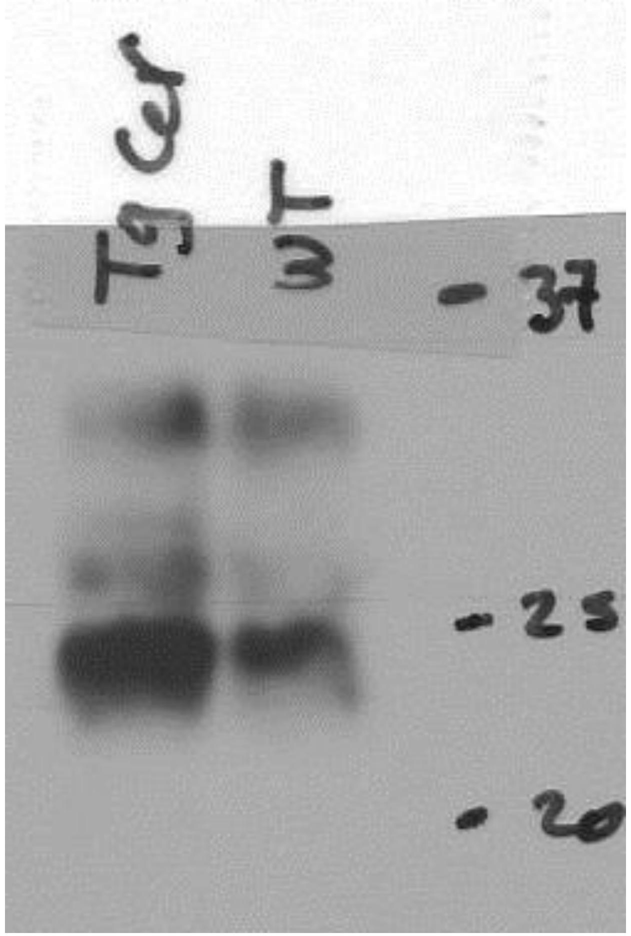

Figure 2a

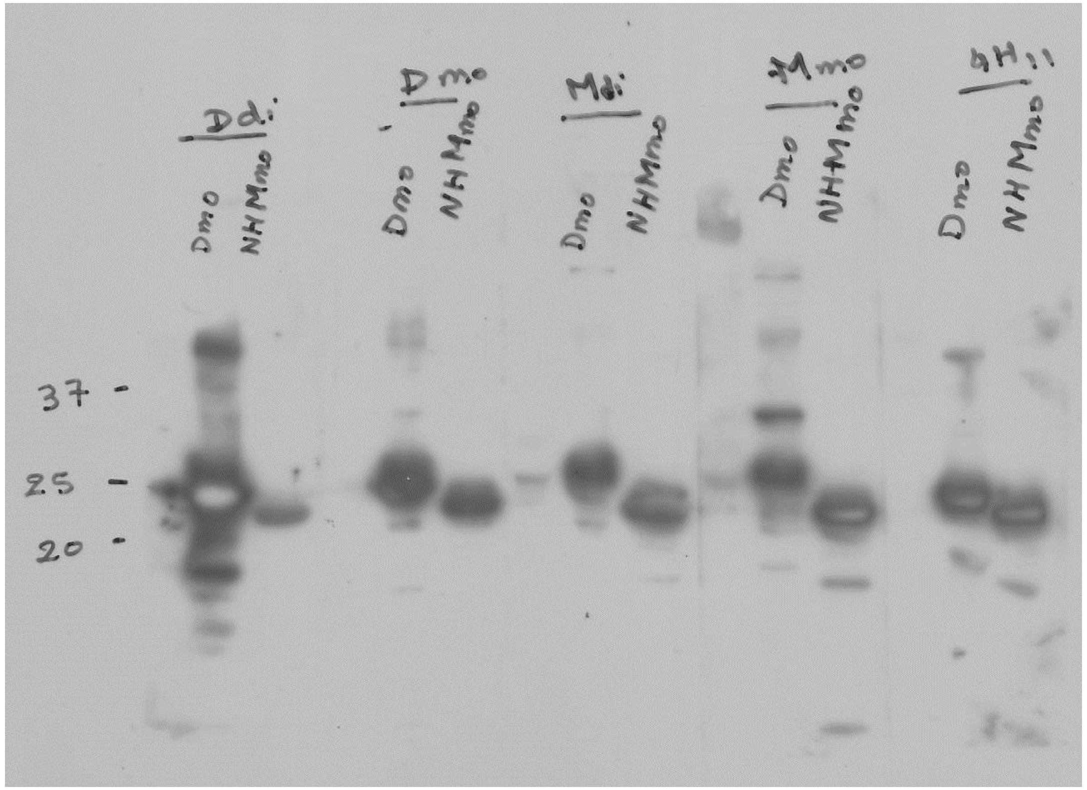

Figure 2c

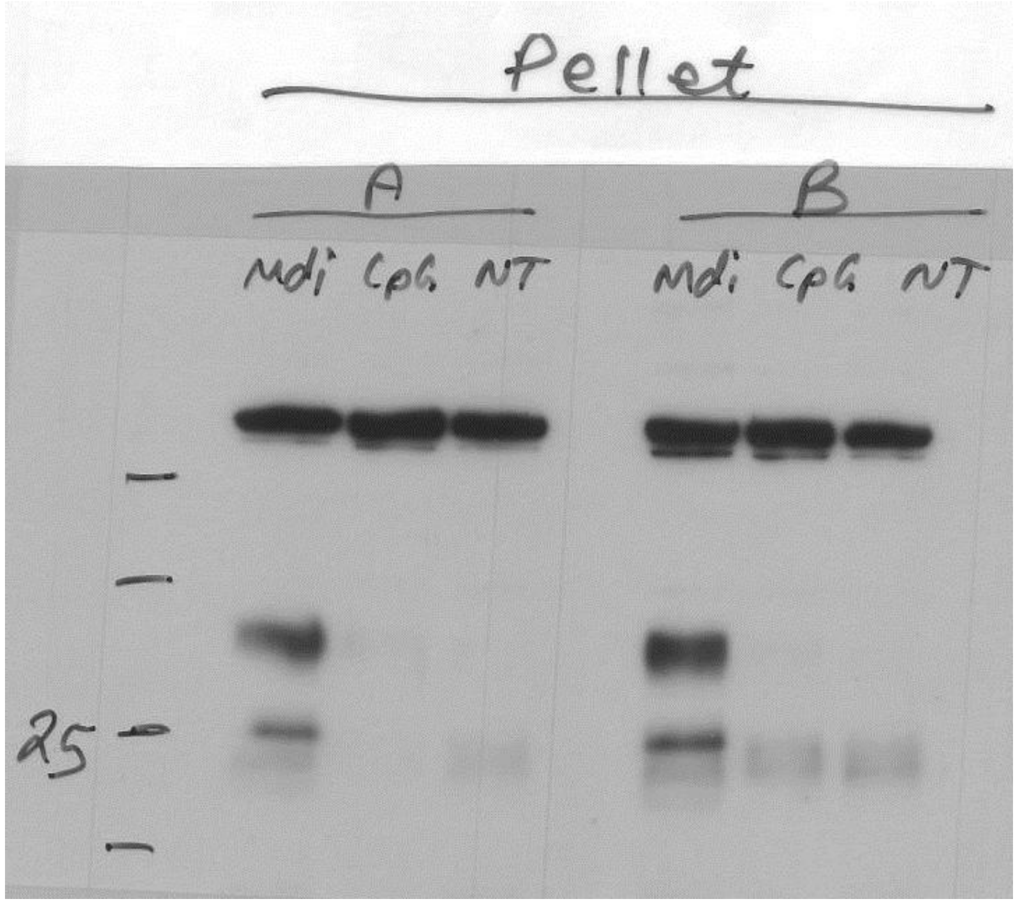

Figure 7a-b

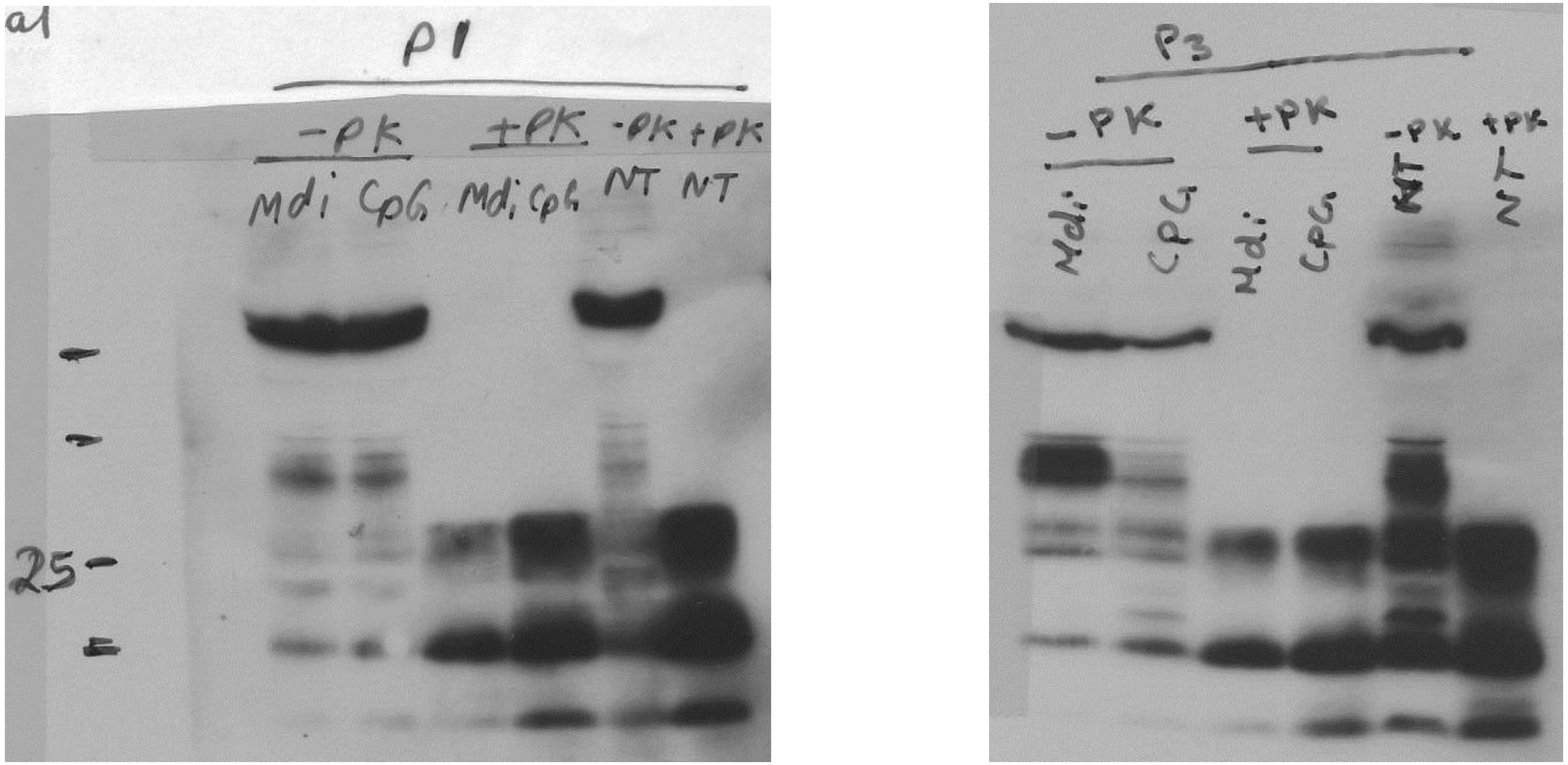
a)
P1
-PK
+PK
-PK +PK
Mdi CpG Mdi CpG NT NT
25
P3
-PK
+PK
-PK +PK
Mdi
CpG
Mdi
CpG
NT
NT

Figure 7 c-d

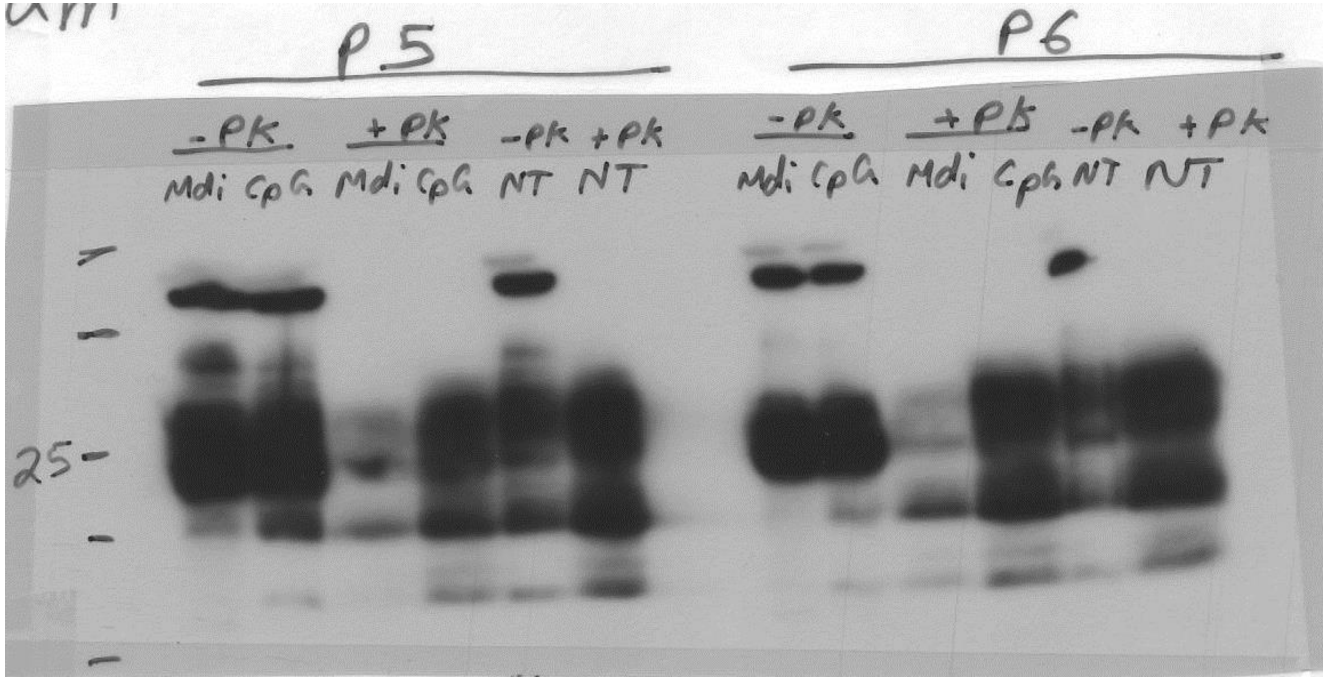
P5
P6
-PK
+PK
-PK +PK
-PK
+PK
-PK
+PK
Mdi CpG Mdi CpG NT NT
Mdi CpG Mdi CpG NT NT
25-

Figure 7 e-h

e f g h

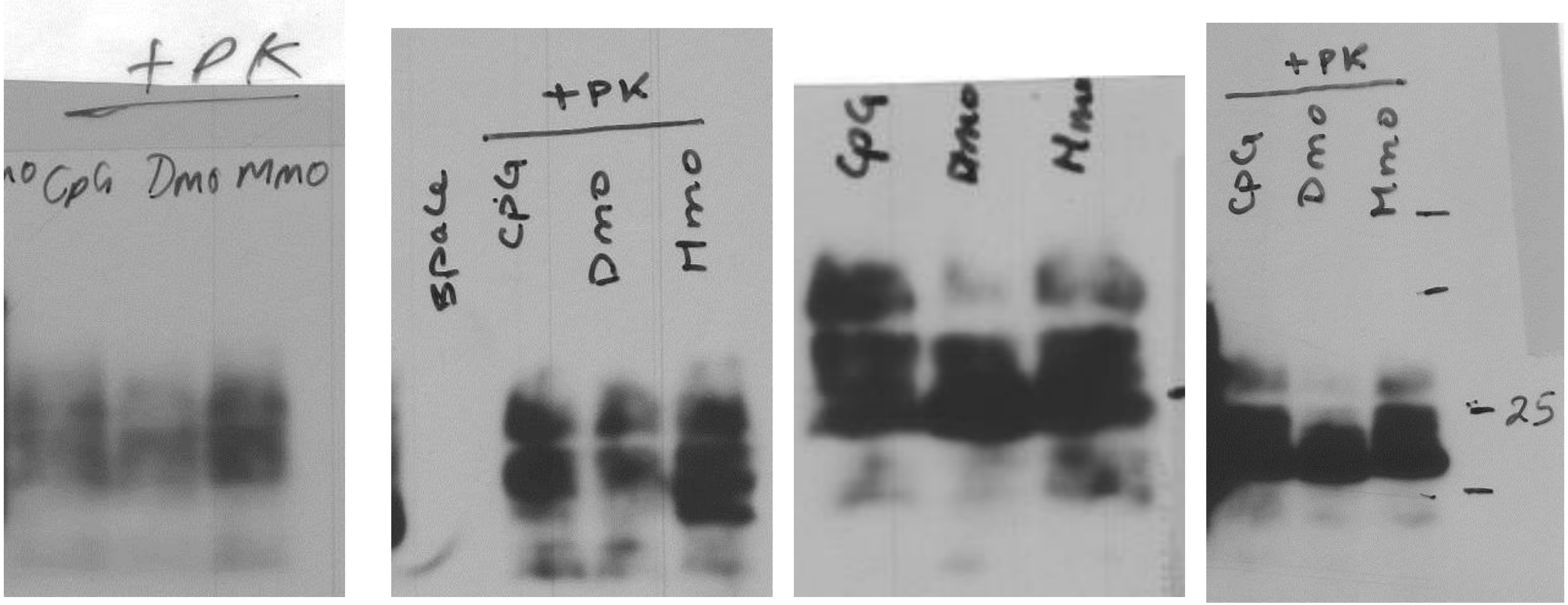

Supplement: Supplementary file 2 — Supplementary Information [file 41598_2017_11235_MOESM2_ESM.pdf]
